# Supplementary material for: Pericytes orchestrate a tumor-restraining microenvironment in glioblastoma
Source: Nat Commun. 2025 Dec 4;16:10918. doi: 10.1038/s41467-025-66985-1 (PMC12680652; doi:10.1038/s41467-025-66985-1)
Supplement: Supplementary file 1 — Supplementary Information [file 41467_2025_66985_MOESM1_ESM.pdf]

Braun, Bolivar et al. 2025.

Pericytes orchestrate a tumor-restraining microenvironment in glioblastoma.

Supplementary Information.

Supplementary Figures.

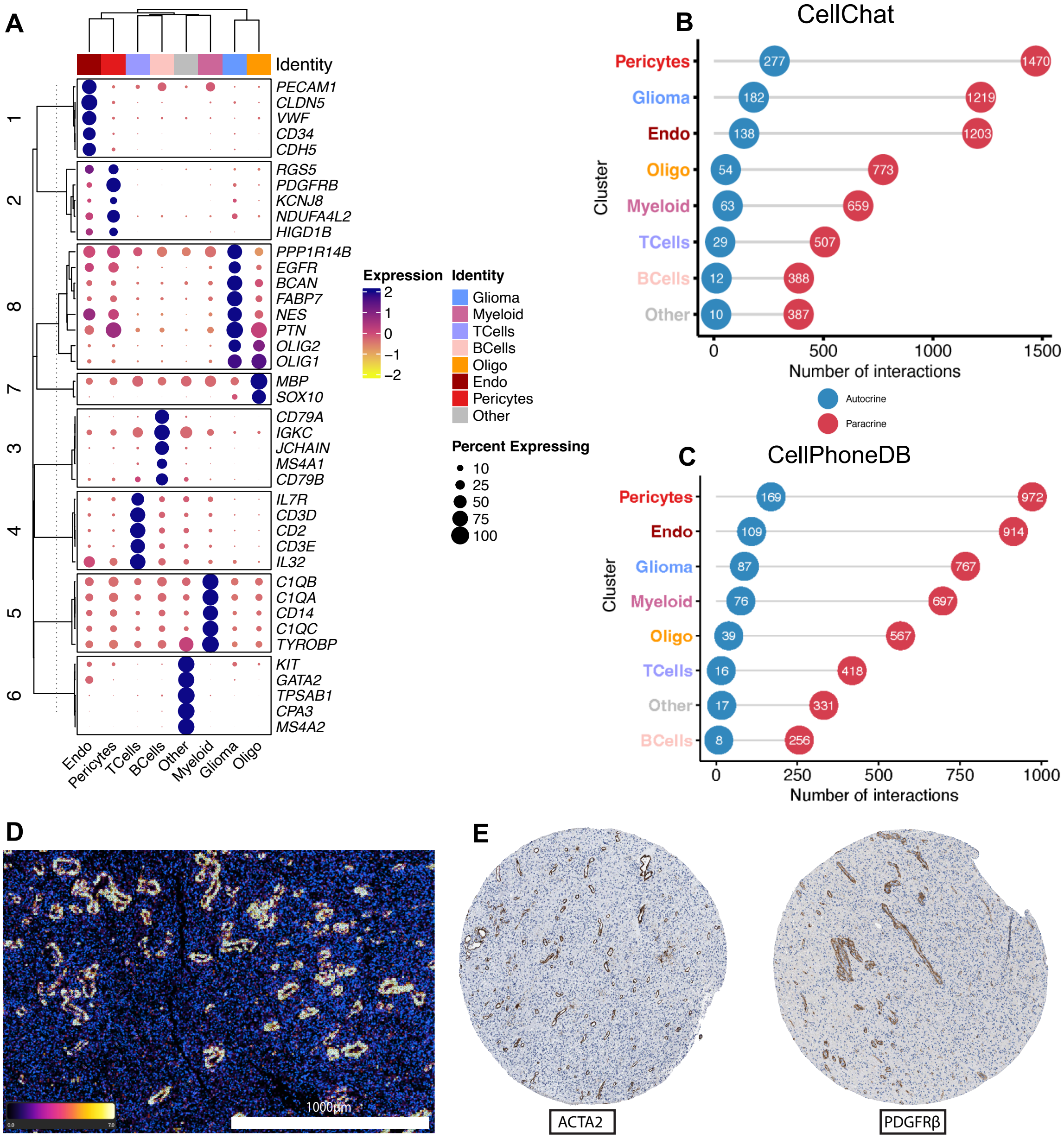

Figure S1. Characterization of different cell groups isolated from human glioma samples, related to Figure 1.

(A) Clustered dot plot showing the scaled average expression of selected top differentially expressed genes across all clusters. Data and annotations from Abdelfattah et al.

(B-C) Lollipop plots showing the number of autocrine and paracrine interactions associated with each cell population for human glioma samples, generated with CellChat (B) and CellPhoneDB (C). Data and annotations from Abdelfattah et al. (2022).

(D) *In situ* gene expression analysis of an adult human GBM tissue, showing nuclear staining (blue), outlined boundaries of cells belonging to putative endothelial clusters (white), and a density map of *PDGFRβ* and *ACTA2*. The dataset was downloaded from the 10x genomics website (see Data availability) and visualized in Xenium explorer (v4.0.0).

(E) TMA of a human HGG (Male, age 48, patient id 3092) immunostained for ACTA2 (left panel) and of a human HGG (Male, age 71, patient id 3091) immunostained for PDGFRβ (right panel). Images are taken from The Human Protein Atlas (see Data availability).

Figure S2

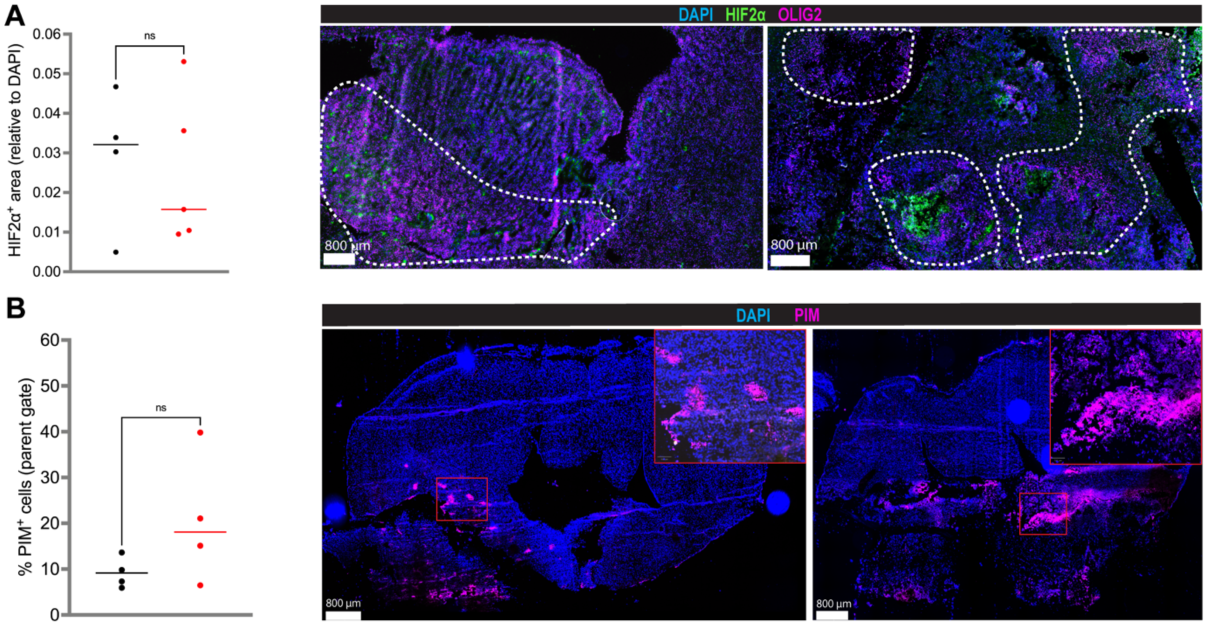

Figure S2. Hypoxia quantification comparing *Pdgfb*$^{ret/+}$ and *Pdgfb*$^{ret/ret}$ murine glioma, related to Figure 2.

(A) Hypoxia analysis (left panel), based on the quantification of HIF2α immunostainings (middle and right panel; OLIG2 immunostaining identifies the tumor borders and infiltrative areas). Dotted white lines represent an estimation of the tumor core areas based on the OLIG2 staining.

Every dot represents the average of several FOVs in one tumor, taken at different positions. Median is shown.

(B) Hypoxia analysis, based on the quantification of PIM^{+} cells with FACS (left panel). Median is shown. The middle and right panel show representative immunostainings of PIM. Boxes denote the enlarged regions.

For plotted data, *Pdgfb*$^{ret/+}$ is depicted in black and *Pdgfb*$^{ret/ret}$ in red.

Two-sided t-test. ns not significant. Source data are provided as a Source Data file.

Figure S3

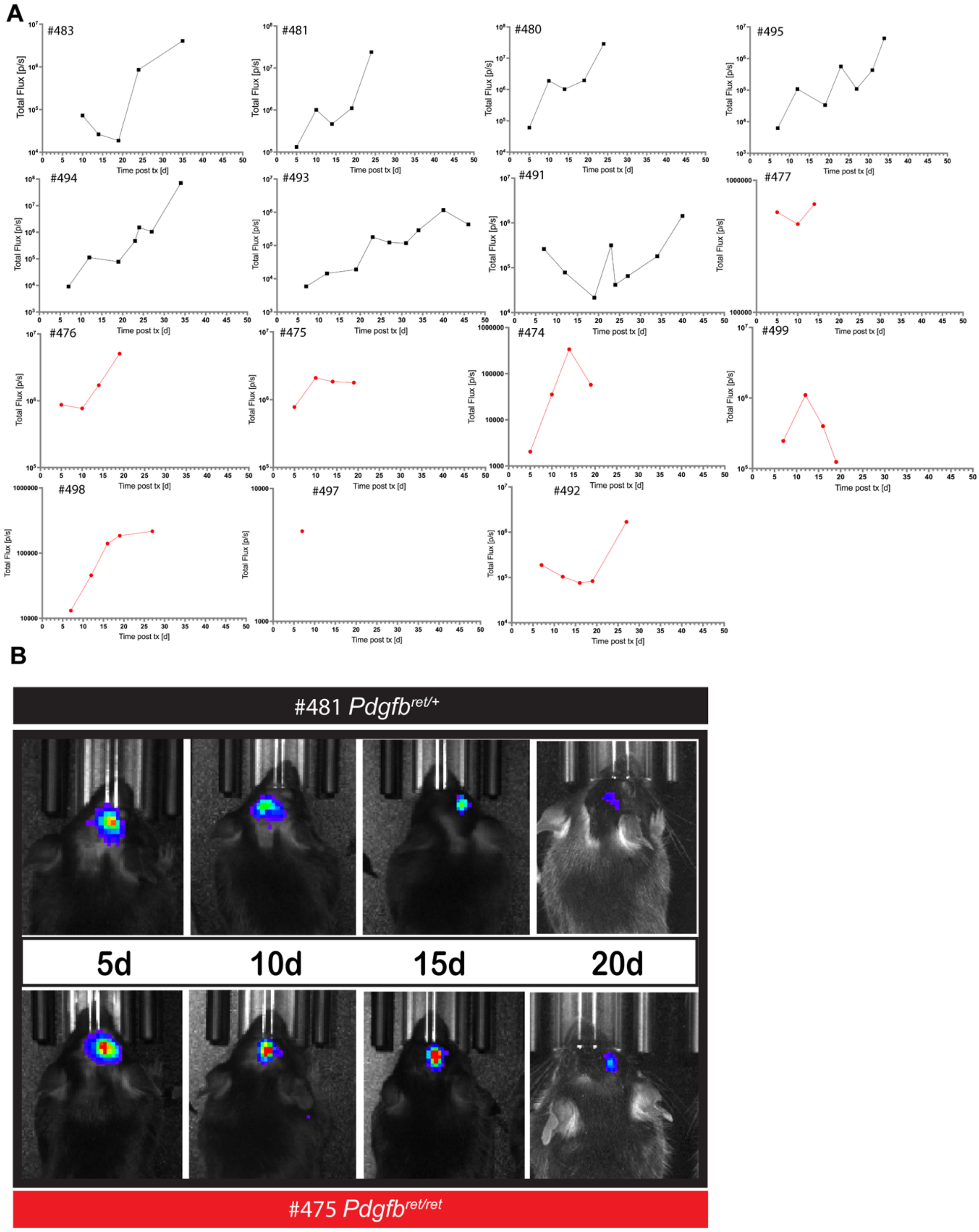

Figure S3. IVIS imaging of tumor bearing mice, related to Figure 2.

(A) Total photon flux for individual *Pdgfb*$^{ret/+}$ and *Pdgfb*$^{ret/ret}$ mice measured during IVIS imaging.

(B) Representative IVIS scans of a pair of *Pdgfb*$^{ret/+}$ and *Pdgfb*$^{ret/ret}$ mice measured over a time course of 5 – 50 days.

Figure S4

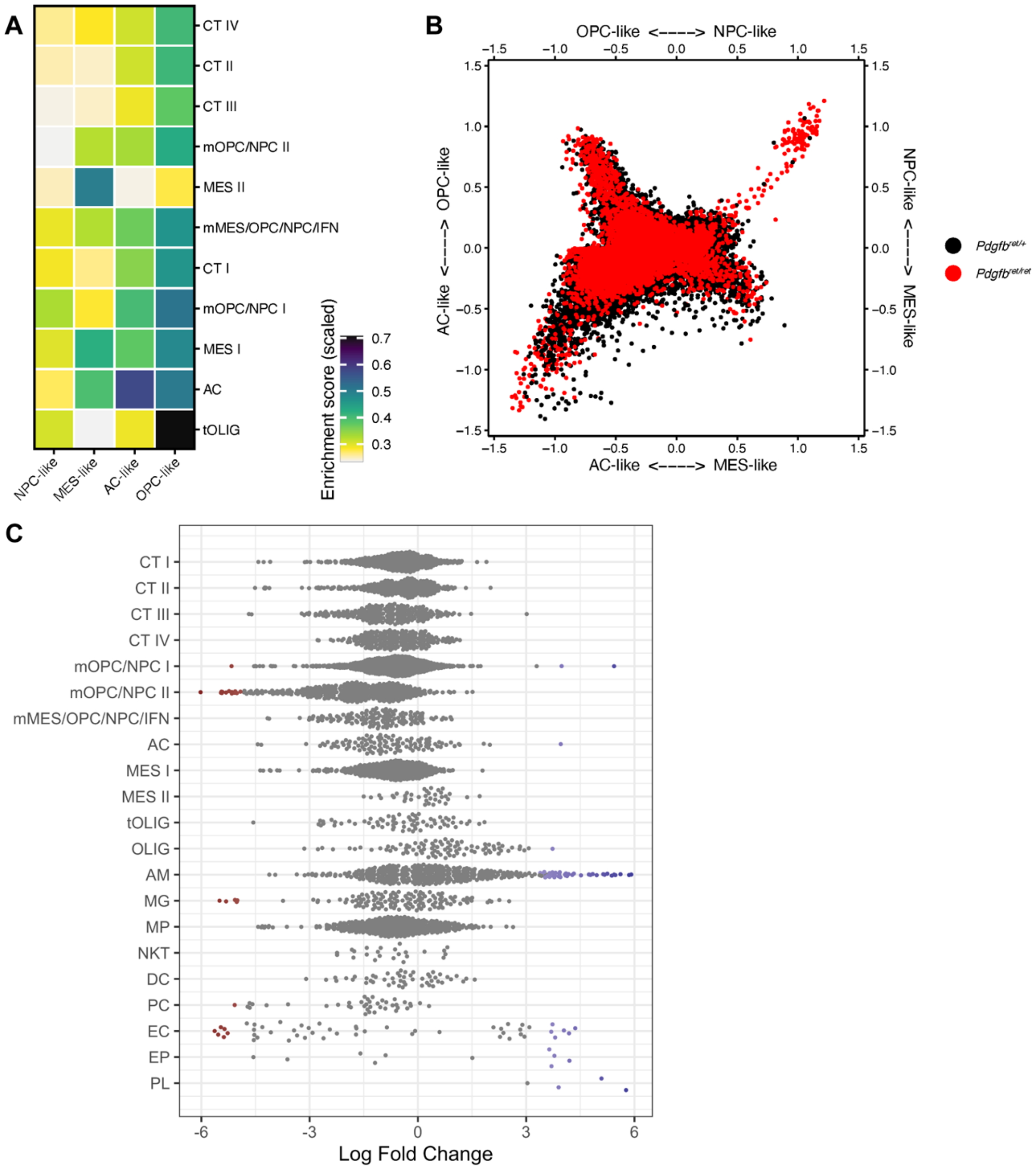

Figure S4. Abundance and distribution of cell populations in RCAS-induced glioma, related to Figure 3.

(A) Heatmap representing the enrichment of the molecular subtype NPC-like, OPC-like, AC-like and MES-like signatures from Neftel et al. in the murine glioma scRNA-seq dataset.

(B) Butterfly plot of the molecular subtype signature scores from Neftel et al. applied to the murine glioma scRNA-seq data, and comparing the relative signature scores of all *Pdgfb*$^{ret/+}$ and *Pdgfb*$^{ret/ret}$ glioma sample cells.

(C) Results from MiloR differential abundance test. Beeswarm plot of the distribution of log fold changes between *Pdgfb*$^{ret/+}$ (reference) and *Pdgfb*$^{ret/ret}$ derived neighborhoods from different cell types. Differential abundance neighborhoods at FDR 20% are colored.

Figure S5

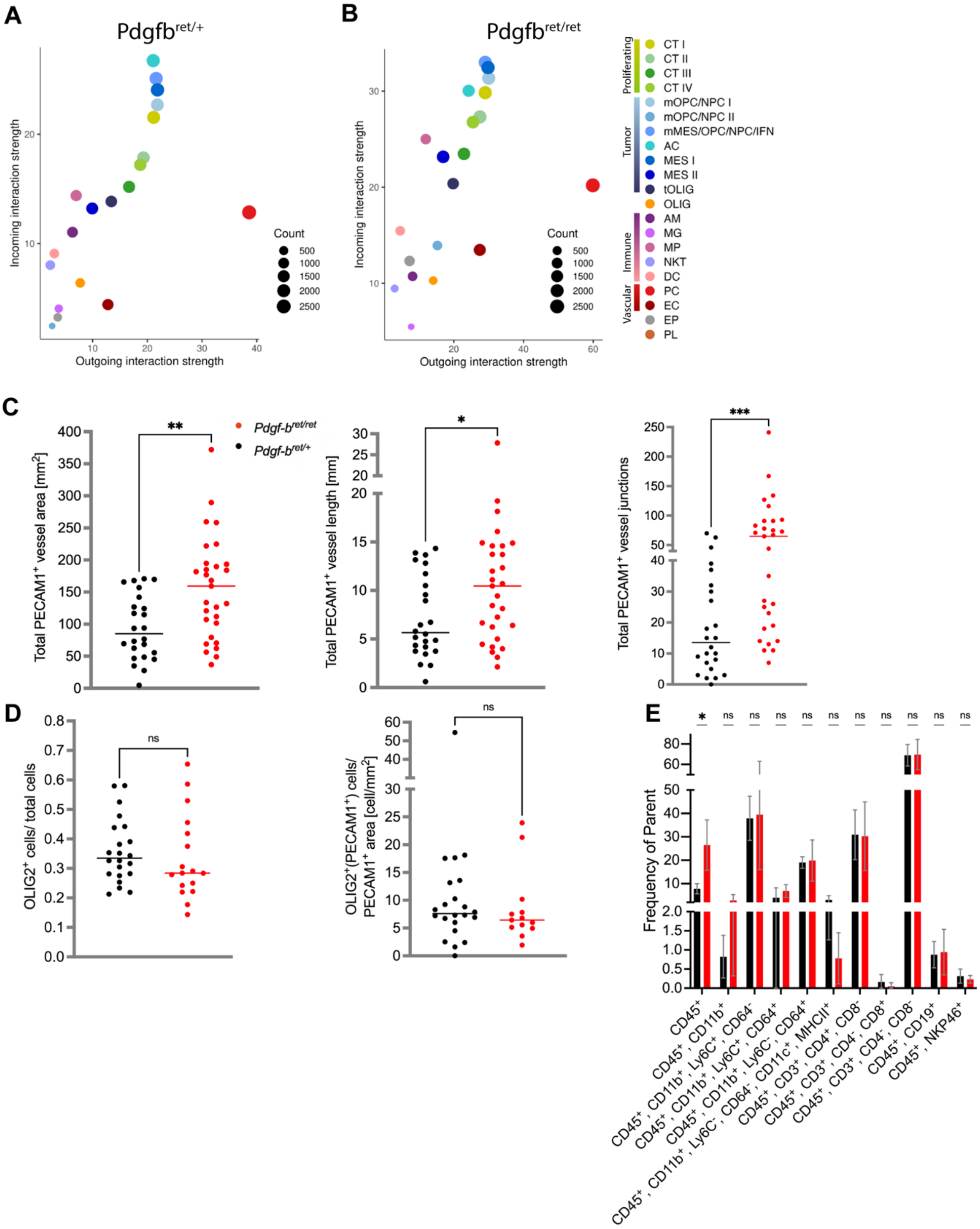

Figure S5. Description of cell communication patterns and tumor cell vascular co-option parameters in pericyte-deprived glioma, related to Figures 3 and 4.

(A-B) Scatter plots showing the total outgoing and incoming interaction strength associated with each cell population for *Pdgfb*$^{ret/+}$ (A) and *Pdgfb*$^{ret/ret}$ (B) glioma derived cells, estimated using CellChat. Dot sizes represent the number of inferred interactions (both incoming and outgoing).

(C) Assessment of glioma tissue parameters total vessel area, length and junctions, comparing *Pdgfb*$^{ret/ret}$ and *Pdgfb*$^{ret/+}$ tissue sections of *p53*$^{-/-}$/*H-Ras* tumor cell induced gliomas. The measurements were done based on immunostaining of PECAM1. Each dot represents the average of several FOVs in one tumor, taken at different positions. Median is indicated.

(D) Quantification of the abundance of OLIG2$^+$ cells (left panel), and OLIG2$^+$ cells in contact with PECAM1$^+$ cells, related to the vessel area (right panel), in the IR. Median is indicated.

(E) FACS-based quantification of different immune cell types in *Pdgfb*$^{ret/+}$ and *Pdgfb*$^{ret/ret}$ gliomas. Mean (bars) with SD is shown.

For plotted data, *Pdgfb*$^{ret/+}$ is depicted in black and *Pdgfb*$^{ret/ret}$ in red.

Two-sided t-test. * p<0.05, ** p<0.01, *** p<0.001, **** p<0.0001, ns not significant.

Source data are provided as a Source Data file.

Figure S6

A

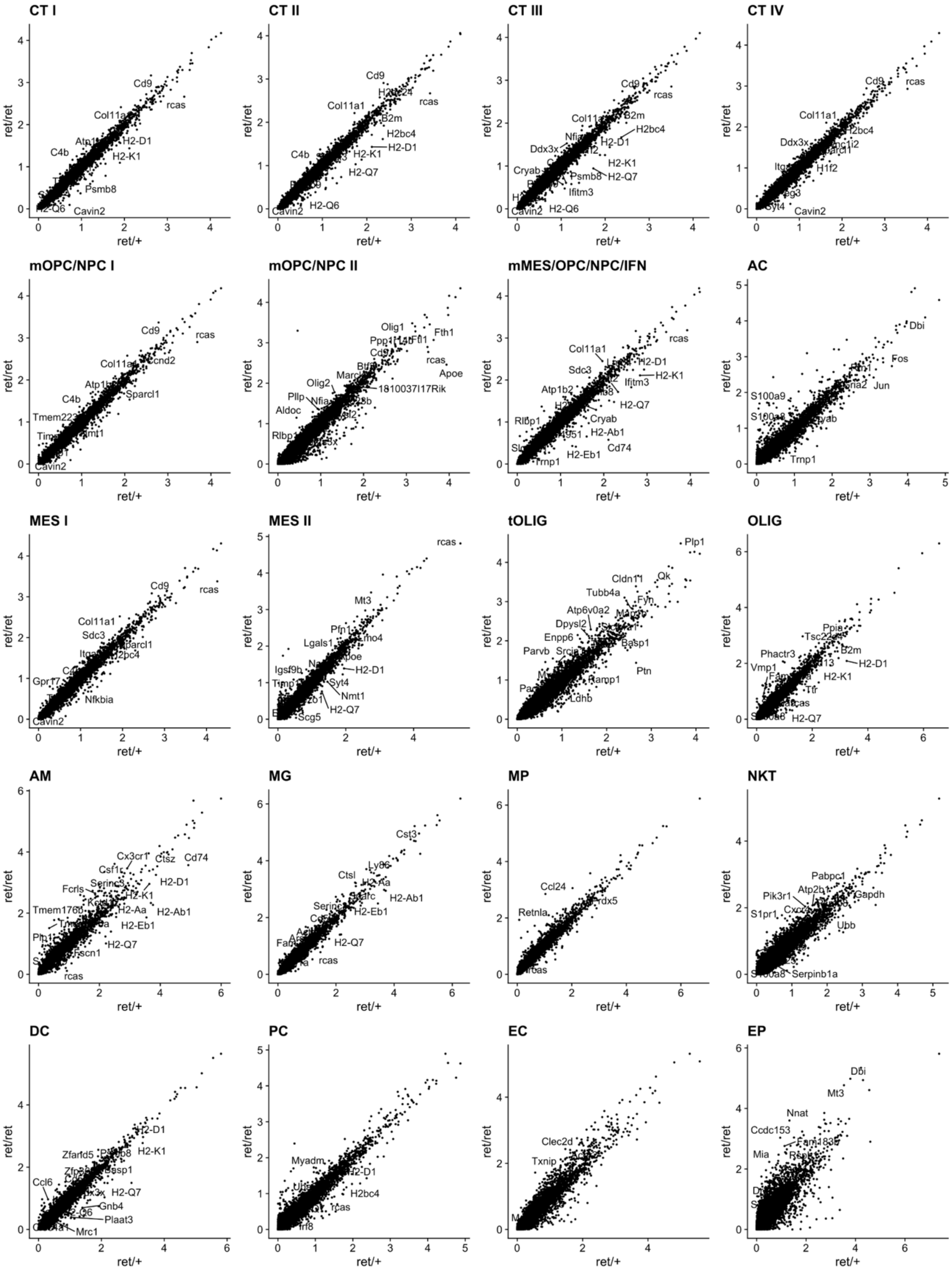

Figure S6. Differential gene expression analyses between *Pdgfb*$^{ret/+}$ and *Pdgfb*$^{ret/ret}$ for each annotated cluster, related to Figure 3.

(A) Scatter plots showing the average expression of each gene in *Pdgfb*$^{ret/+}$ and *Pdgfb*$^{ret/ret}$ glioma derived cells. The top 20 differentially expressed genes with log2 fold change of at least 0.5 between conditions are named. Source data are provided as a Source Data file.

Figure S7

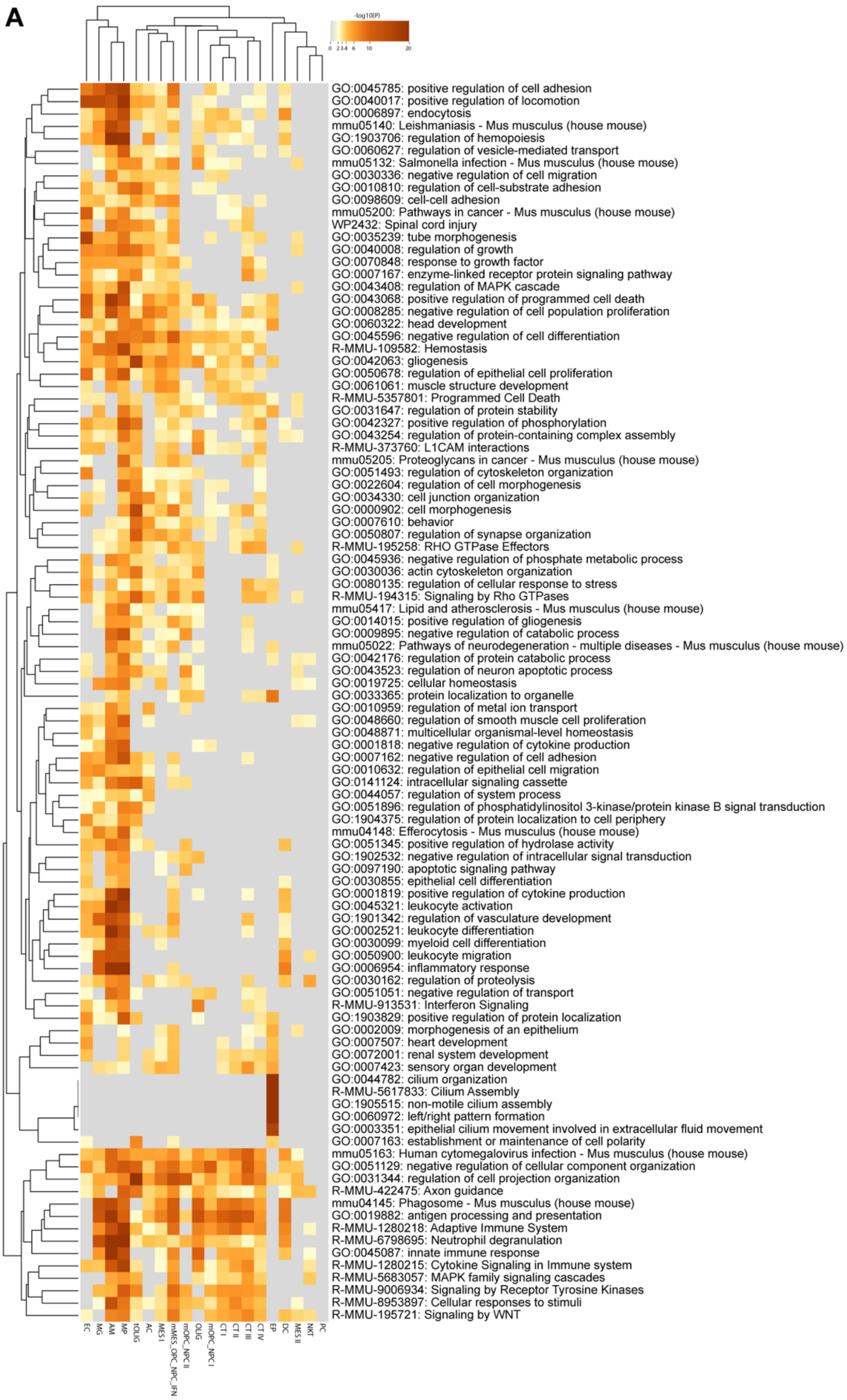

Figure S7. GO overrepresentation analysis between *Pdgfb$^{ret/+}$* and *Pdgfb$^{ret/ret}$* tumors, related to Figure 4.

(A) Heatmap of the top 100 enriched GO terms across DEG between *Pdgfb$^{ret/+}$* and *Pdgfb$^{ret/ret}$* tumors in different clusters as inferred by Metascape, colored by p-values.

Figure S8

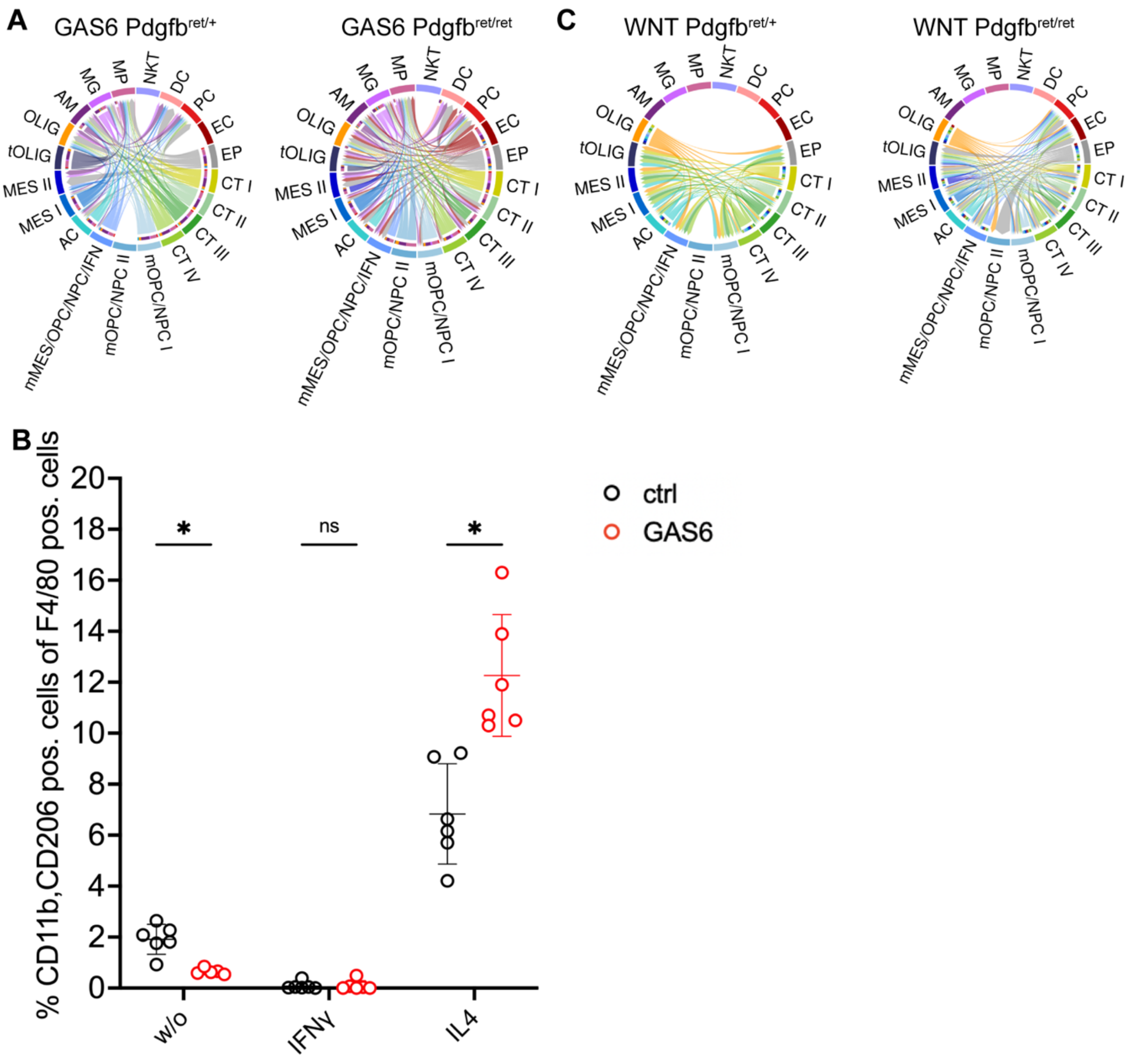

Figure S8. Comparison of cell-cell-interactions between *Pdgfb*$^{ret/+}$ and *Pdgfb*$^{ret/ret}$ tumor and stromal cells, related to Figure 4.

(A) Chord diagram of GAS6 signaling pathway that shows differences between *Pdgfb*$^{ret/ret}$ and *Pdgfb*$^{ret/+}$ tumors, performed with CellChat.

(B) FACS-based quantification of CD206^{+} murine, bone marrow derived, proliferating macrophages, cultured with GAS6 (red open circles) or mock (black open circles). In addition, either IL4, IFNγ or no cytokine was added to the cultures. Analysis was performed 72 h post addition of the respective compounds. Two-sided t-test. Mean with SD is shown.

(C) Chord diagram showing different activity of WNT signaling in *Pdgfb*$^{ret/ret}$ and *Pdgfb*$^{ret/+}$ tumors, performed with CellChat.

* $p<0.05$, ns not significant

Figure S9

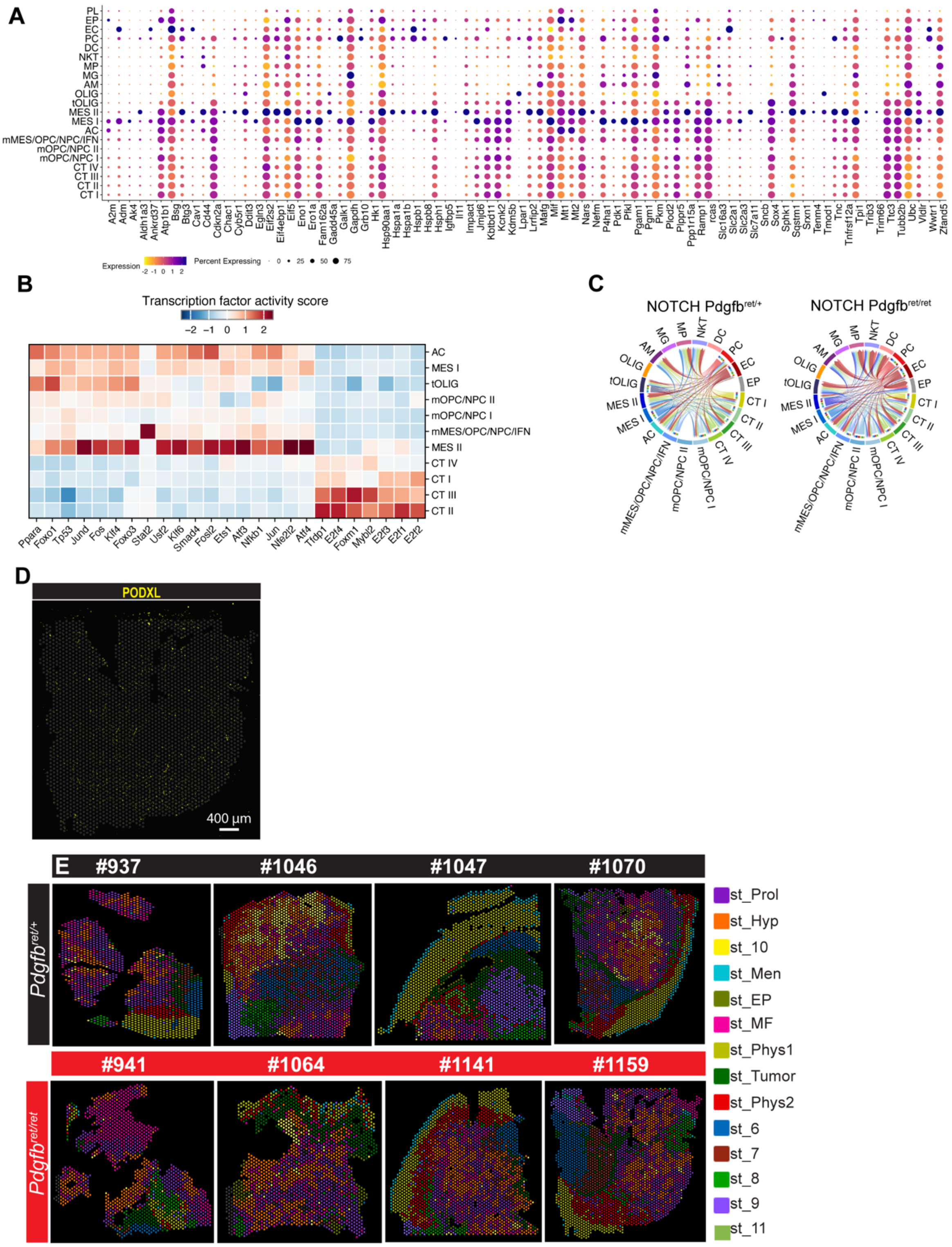

Figure S9. Validation and integration of the scRNA-seq and stRNA-seq data, related to Figure 5.

(A) Dot plot showing the scaled expression of the MES I and MES II top 50 differentially expressed genes (combined *Pdgfb*$^{ret/+}$ and *Pdgfb*$^{ret/ret}$) across all scRNA-seq clusters, that are not shown in Figure 5A.

(B) Transcription factor activity analysis of the scRNA-seq data using DoRothEA. The heatmap represents the top 25 transcription factor activity scores among the identified cell groups.
(C) Chord diagram of the NOTCH signaling pathway, comparing its activity in *Pdgfb*$^{ret/ret}$ and *Pdgfb*$^{ret/+}$ tumors, performed with CellChat.
(D) VGES capture area of stRNA-seq sample #1159, showing an immunostaining of PODXL (this panel is complementary to Figure 5B).
(E) VGES capture area images of 4 *Pdgfb*$^{ret/+}$ (upper panels, black label) and 4 *Pdgfb*$^{ret/ret}$ (bottom panels, red label) glioma sections, indicating 14 spatial clusters, identified based on the integration of all 8 samples, using Harmony.
Source data are provided as a Source Data file.

Figure S10

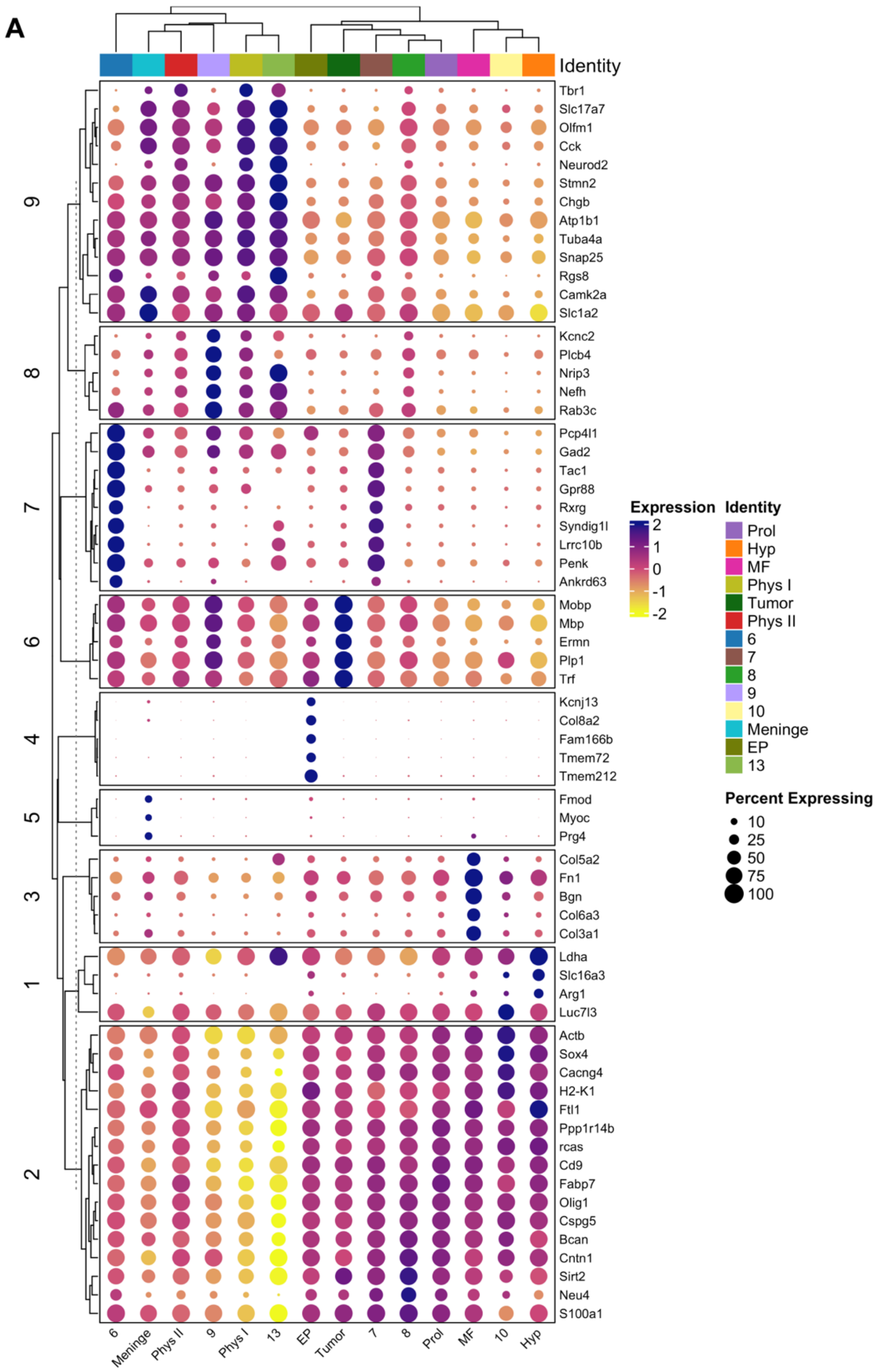

Figure S10. Clustered dot plot based on selected DEG of the stRNA-seq data, related to Figure 5.

(A) Clustered dot plot showing the scaled average expression of selected top DEG across all stRNA-seq clusters. Source data are provided as a Source Data file.

Figure S11

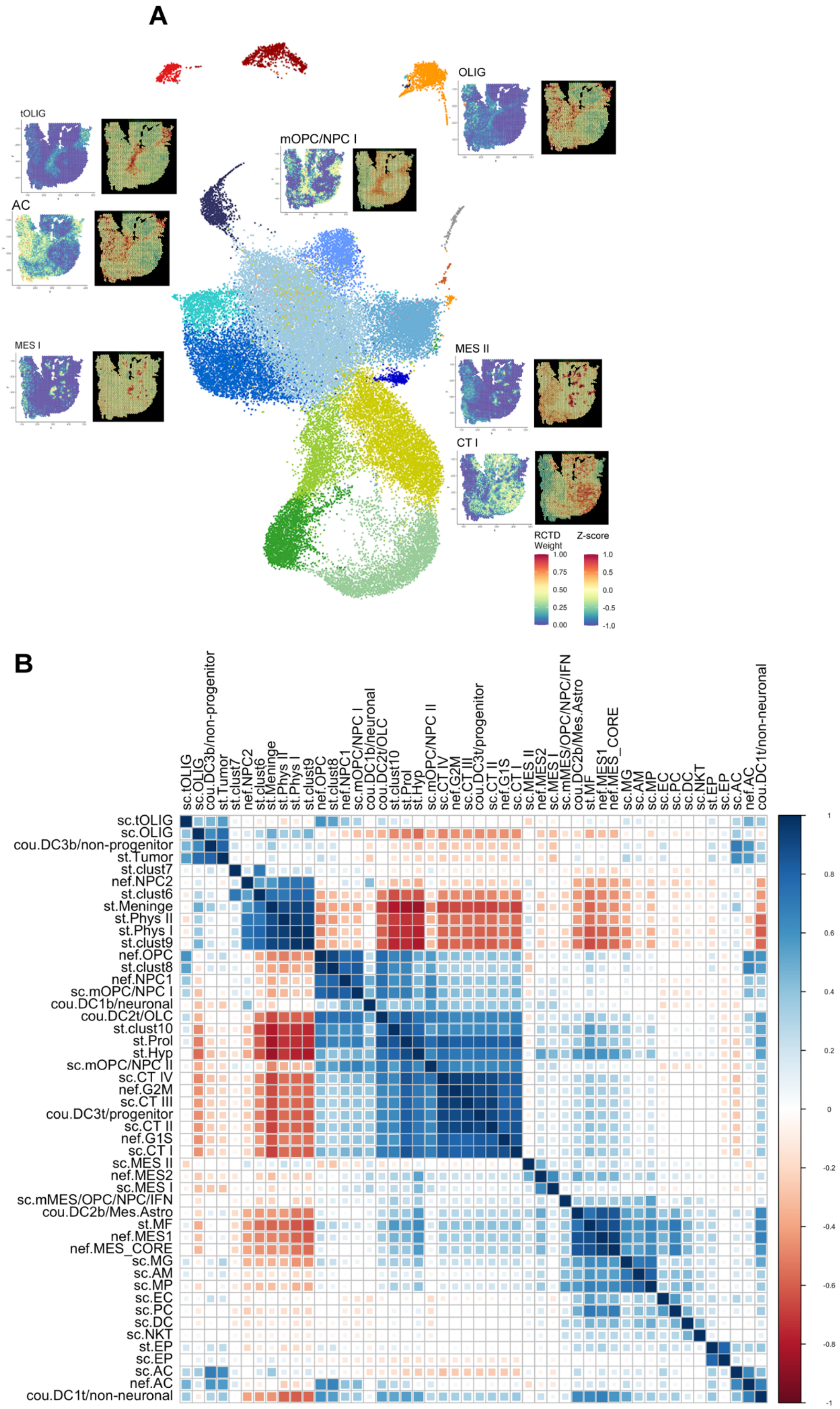
A
tOLIG
OLIG
mOPC/NPC I
AC
MES I
MES II
CT I
RCTD Weight
Z-score
1.00
0.75
0.50
0.25
0.00
1.0
0.5
0.0
-0.5
-1.0
B
sc.tOLIG
sc.OLIG
cou.DC3b/non-progenitor
st.Tumor
st.clust7
nef.NPC2
st.clust6
st.Meninge
st.Phys II
st.Phys I
st.clust9
nef.OPC
st.clust8
nef.NPC1
sc.mOPC/NPC I
cou.DC1b/neuronal
cou.DC2t/OLC
st.clust10
st.Prol
st.Hyp
sc.mOPC/NPC II
sc.CT IV
nef.G2M
sc.CT III
cou.DC3t/progenitor
sc.CT II
nef.G1S
sc.CT I
sc.MES II
nef.MES2
sc.MES I
sc.mMES/OPC/NPC/IFN
cou.DC2b/Mes.Astro
st.MF
nef.MES1
nef.MES_CORE
sc.MG
sc.AM
sc.MP
sc.EC
sc.PC
sc.DC
sc.NKT
st.EP
sc.EP
sc.AC
nef.AC
cou.DC1t/non-neuronal
1
0.8
0.6
0.4
0.2
0
-0.2
-0.4
-0.6
-0.8
-1

Figure S11. Joint visualization of the scRNA-seq and stRNA-seq data, related to Figure 5.

(A) UMAP plot of scRNA-seq dataset, together with the VGES of stRNA-seq sample #1159, colored by mean Z-scores (right panels), and normalized RCTD weights (left panels) of selected scRNA-seq cell cluster signatures.

(B) Clustered correlation matrix showing pairwise Pearson's correlation coefficients of the mean Z-scores from signatures of different cell identity or spatial transcriptomics clusters from the scRNA-seq and stRNA-seq datasets from this study, Neftel et al. (nef) and Couturier et al. (cou).

Figure S12

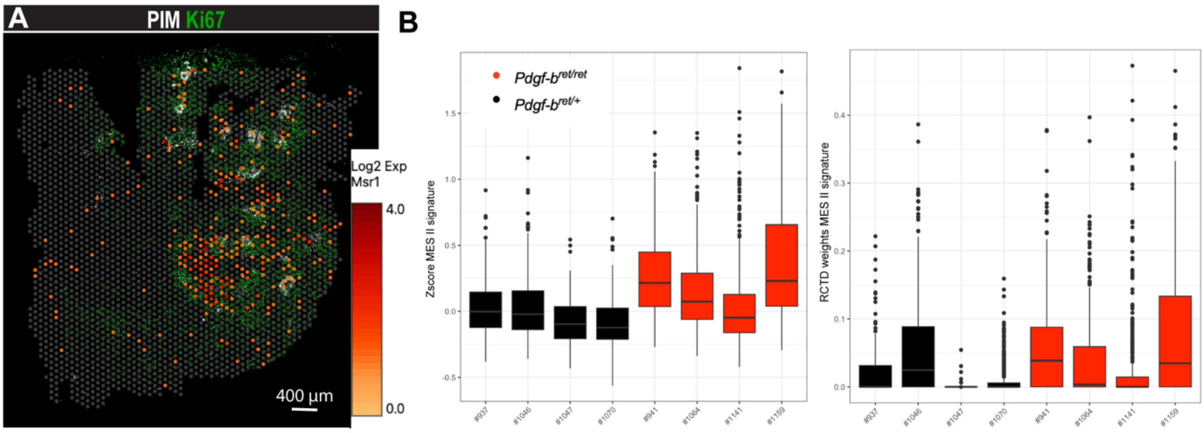

Figure S12. Spatial analysis of *Pdgfb*$^{ret/+}$ and *Pdgfb*$^{ret/ret}$ tumors, related to Figure 5.

(A) VGES capture area of stRNA-seq sample #1159, showing the logNormalized expression of *Msr1*, together with immunostaining of Ki67 and PIM.

(B) Boxplots showing the MES II signature Z-scores (left panel) and inferred proportion of MES II cells by RCTD (right panel) for the st_Hyp clusters of the *Pdgfb*$^{ret/+}$ (black bars) and *Pdgfb*$^{ret/ret}$ (red bars) Visium samples. Boxplots show the median (centre line), the 25th and 75th percentiles (bounds of box), and whiskers extending to data points within 1.5 × the interquartile range; points outside this range are shown as outliers.

Figure S13

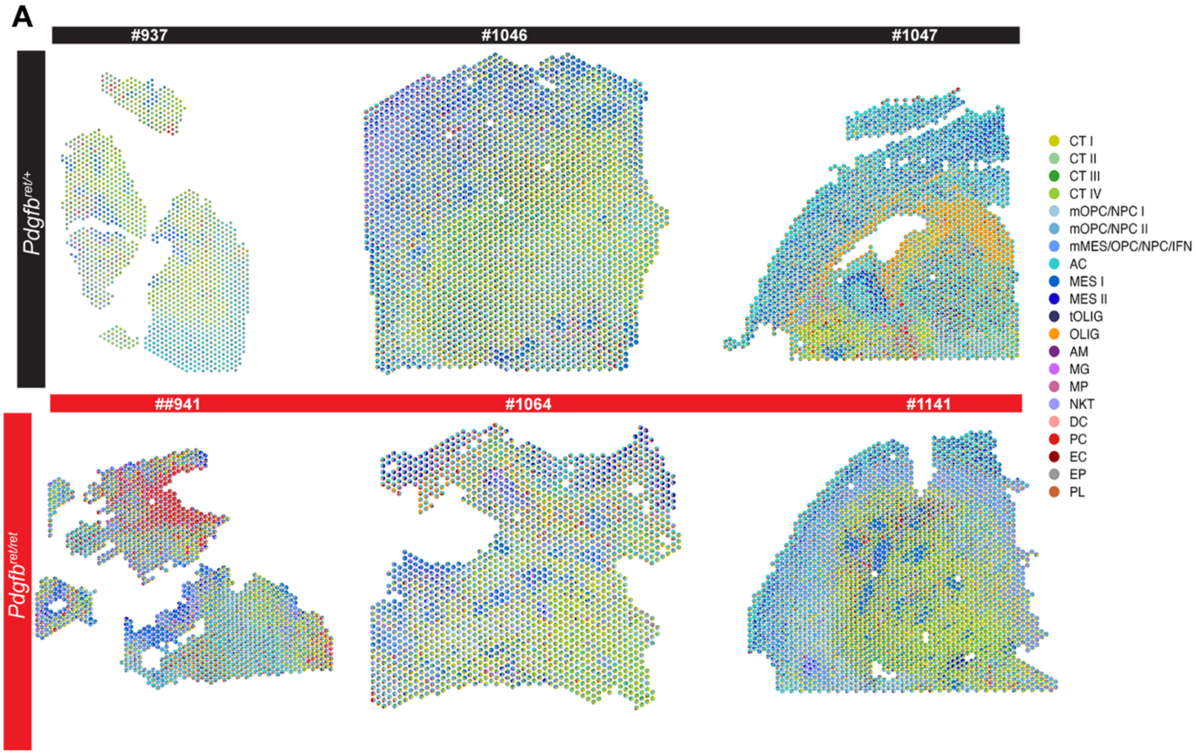

Figure S13. Capture spot deconvolution, related to Figure 6.

(A) Spatial scatterpie charts of *Pdgfb*$^{ret/+}$ (black label) and *Pdgfb*$^{ret/ret}$ (red label) glioma sections showing the RCTD proportions of different cell types per spot.

Figure S14

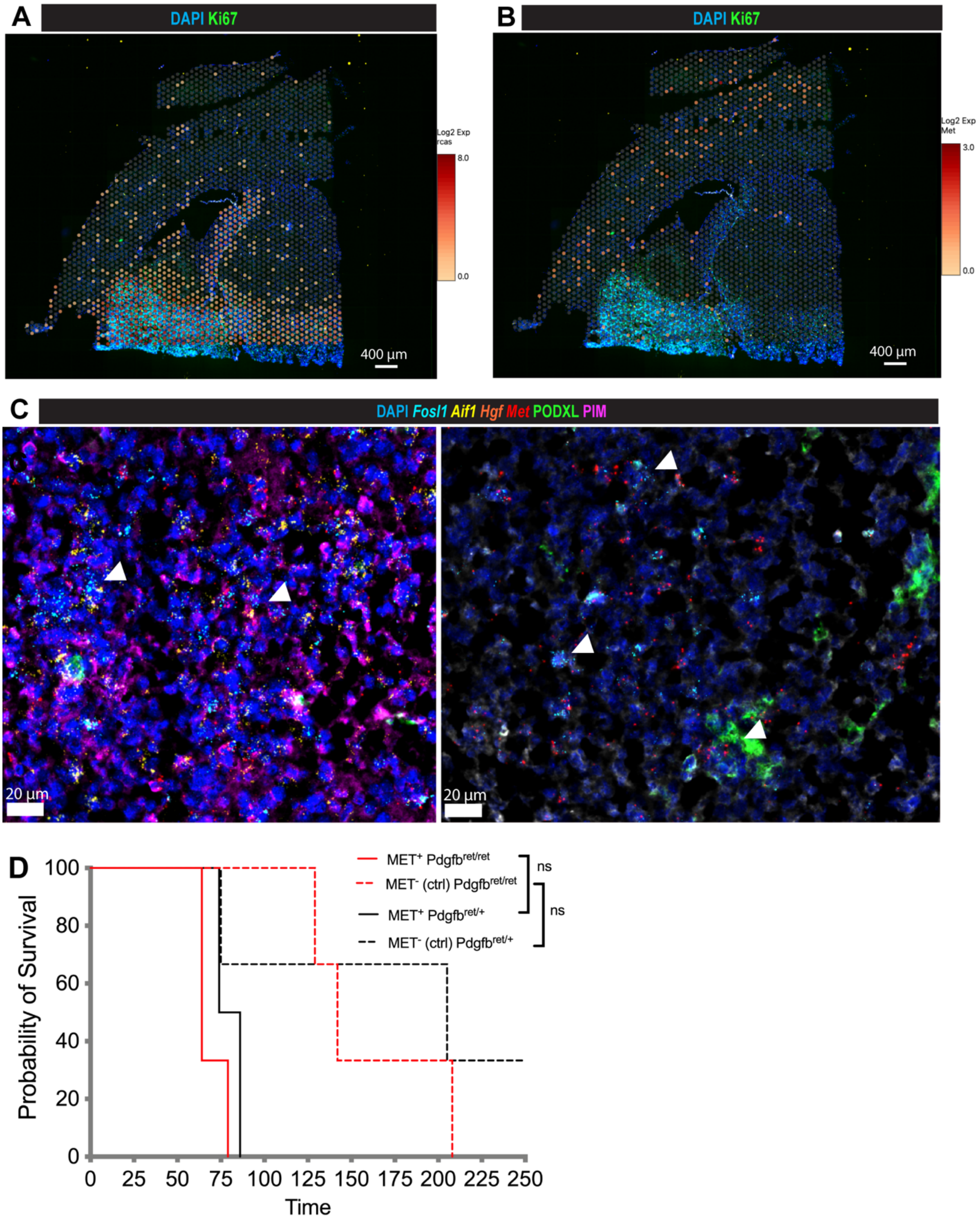

Figure S14. Localization and *in vivo* assay of MES II cells, related to Figure 7.

(A-B) VGES capture area of stRNA-seq sample #1047, showing the expression of an RCAS unique sequence (A) and *Met* (B), together with immunostaining of Ki67 and PIM.

(C) Combined multiplexed immunostaining-ISH analysis for the detection of *Hgf*, *Aif1*, *Fosl1*, *Met*, PODXL and PIM in PDGFB-induced gliomas, derived from *Pdgfb*$^{ret/ret}$ mice. Arrow heads indicate *Fosl1*$^{+}$ cells.

(D) Kaplan-Meier curves showing symptom-free survival of mice transplanted with MET^{+} and MET^{-} glioma cells, stratified into *Pdgfb*$^{ret/ret}$ and *Pdgfb*$^{ret/+}$ mouse groups. n = 3 for all groups except for MET+ *Pdgfb*$^{ret/ret}$ were n = 2. Log-rank test. ns not significant

Figure S15

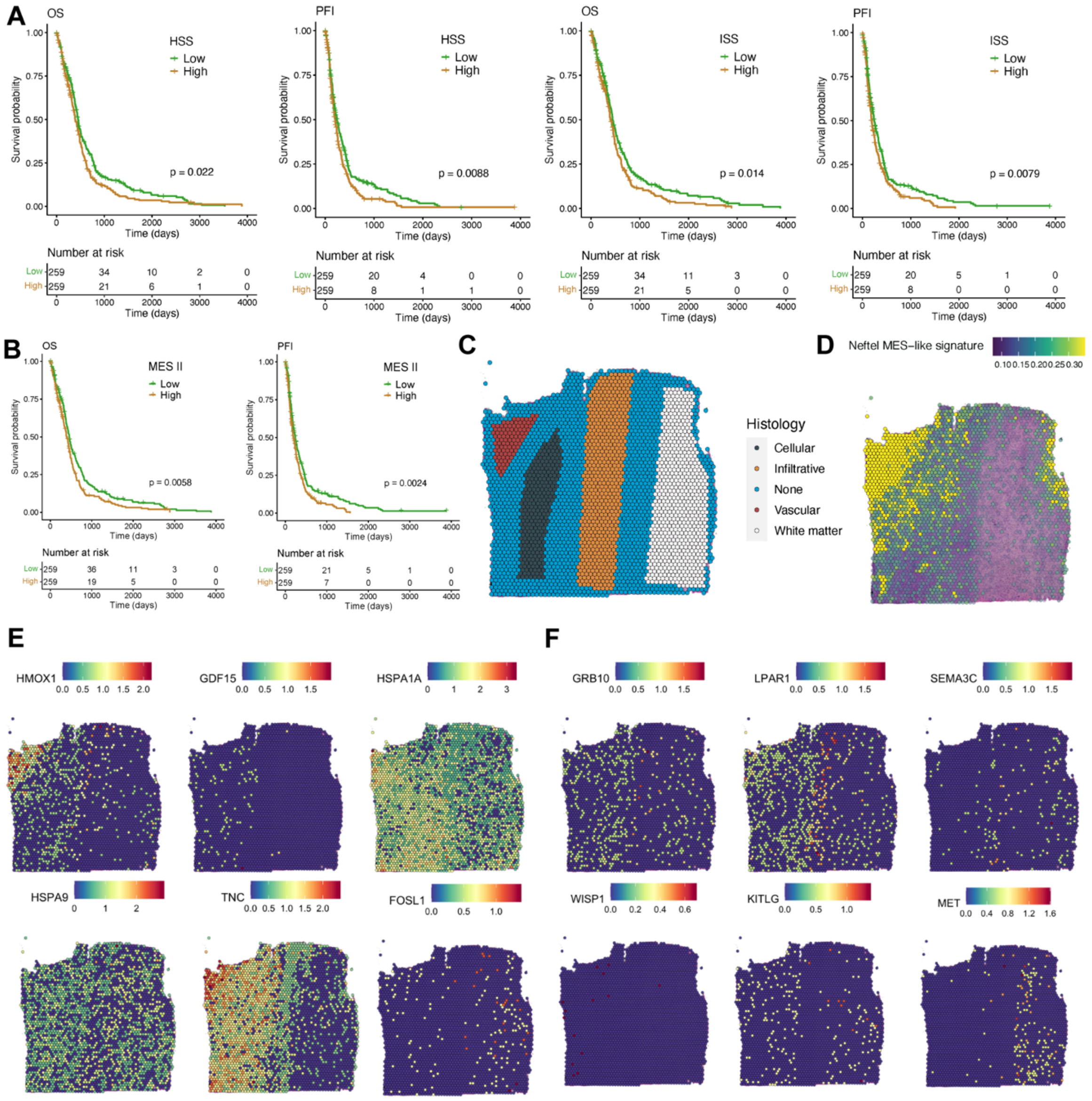

Figure S15. Spatial characterization of GBM patient samples, based on histopathological and transcriptomics analysis, related to Figure 8.

(A) The Kaplan-Meier curves show the overall survival (OS) and progression-free interval (PFI) probabilities of the high (brown) and low (green) HSS and ISS expression groups in the TCGA GBM cohort (n = 518). p-value: log-rank test.

(B) The Kaplan-Meier curves show the OS and PFI probabilities of the high (brown) and low (green) MES II expression group in the TCGA GBM cohort

(n = 518). log-rank test.

(C) Spatial plot showing the histological morphology assessment in sample #UKF269_T made by Ravi et al.

(D) Spatial plot and heatmap showing the MES-like (Neftel et al.) module scoring in sample #UKF269_T.

(E-F) Spatial plots showing the expression of HSS genes (E) and ISS genes (F) in sample #UKF269_T.

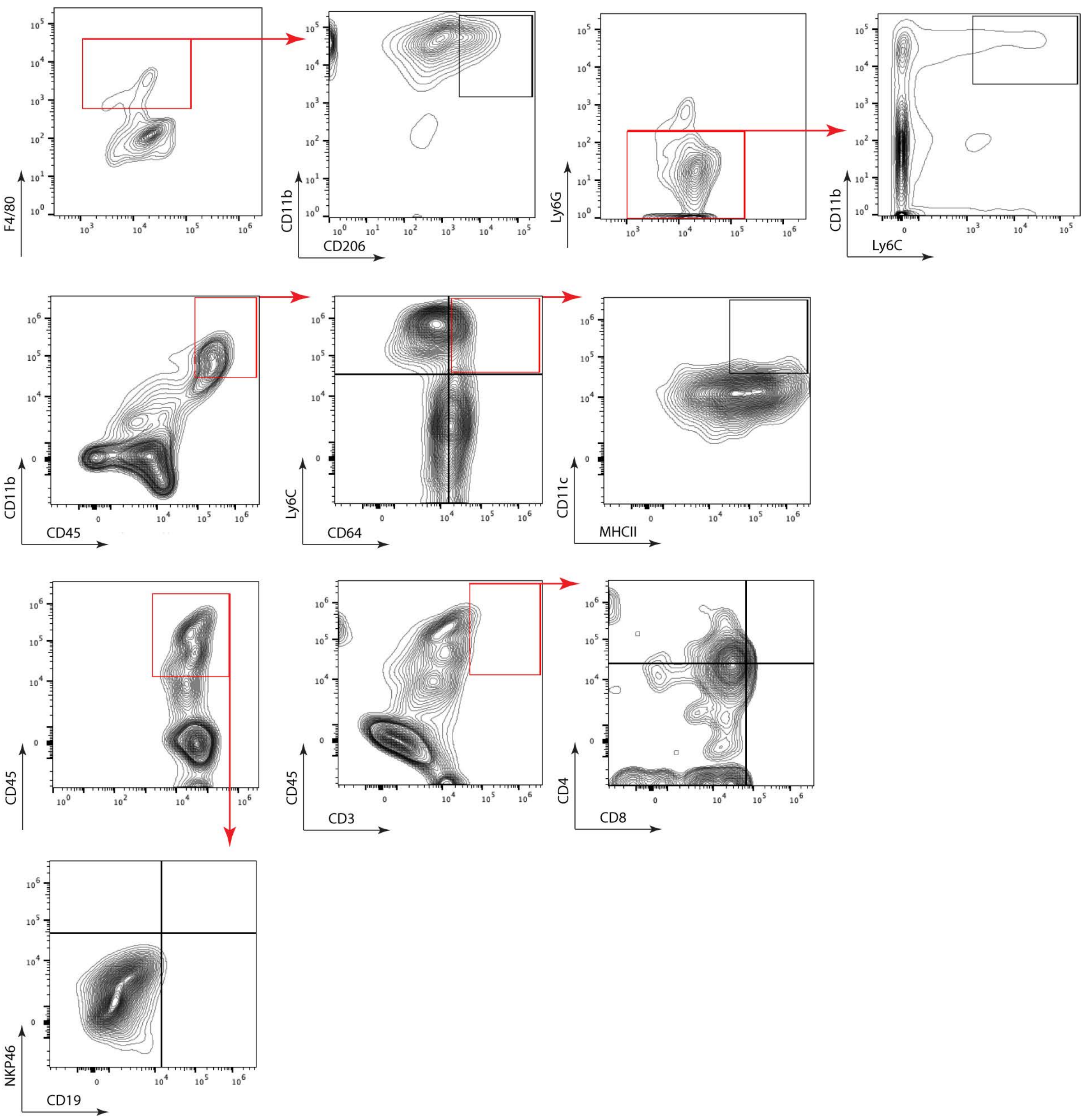

Figure S16. FACS gating strategy.

Resource Tables

REAGENT or RESOURCE	SOURCE	IDENTIFIER
**Antibodies and probes**		
anti-human/mouse CD11b-APC-Cy7 (clone M1/70)	BD Pharmingen	cat# 557657
anti-mouse CD206 (clone MR5D3)	BioRad	cat# MCA2235GA
anti-mouse CD206-APC (clone C068C2)	BioLegend	cat# 141708
anti-mouse CD45-AF700 (clone 30-F11)	BioLegend	cat# 103127
anti-mouse CD3e-BV711 (clone 145-2C11)	BD Pharmingen	cat# 5631123
anti-mouse CD19-APC (clone 6D5)	BioLegend	cat# 115511
anti-mouse CD4-BV786 (clone GK1.5)	BD Pharmingen	cat# 563331
anti-mouse CD8-FITC (clone 53-6.7)	Biolegend	cat# 100705
anti-mouse CD335BV421 (clone 29A1.4)	BioLegend	cat# 137611
anti-mouse CD25-PE-Cy7 (clone PC61)	BD Pharmingen	cat# 561780
anti-mouse CD11b-BV605 (clone M1/70)	BD Pharmingen	cat# 563015
anti-mouse CD11c-FITC (clone N418)	BioLegend	cat# 117305
anti-mouse MHCII-PE-Cy7 (clone M5/114.15.2)	BioLegend	cat# 107629
anti-mouse CD64-APC (clone X54-5/7.1)	BioLegend	cat# 139305
anti-mouse CD103-APC-Cy7 (clone 2E7)	BioLegend	cat# 121431
anti-mouse Ly-6G-BV785 (clone 1A8)	BioLegend	cat# 127645
anti-mouse Ly-6C-BV421 (clone HK1.4)	BioLegend	cat# 128031
anti-mouse PECAM-1 (CD31) (1:400; clone Mec13.3)	BD Pharmingen	cat# ab131279
anti-mouse CD44 (clone X3)	BD Pharmingen	cat# 550538
anti-mouse F4/80-FITC (clone BM8)	BioLegend	cat# 123107
anti-mouse HIF2α	Novus Biologicals	cat# NB100-132
anti-mouse KI67 (SolA15)	eBioscience	cat# 14-5698-82
anti-mouse Ly-6C-PE (clone HK1.4)	eBioscience	cat# 12-5932-80
anti-mouse Ly-6G-PE-Cy7 (clone 1A8)	BD Pharmingen	cat# 560601
anti-mouse Mab (clone 11.23.22.r)	hpi	cat# HP1-XXXX
anti-mouse Mab-FITC (clone 11.23.22.r)	hpi	cat# FITC-Mab
anti-mouse OLIG2	Millipore	cat# AB9610
anti-mouse OLIG2 (clone SP07-02)	Invitrogen	cat# MA5-32039
anti-mouse PDGFR (clone PR7212)	R&D Systems	cat# AF1042
anti-mouse Podocalyxin	R&D Systems	cat# AF1556
anti-mouse Smooth muscle actin (clone 1A4)	Sigma-Aldrich	cat# A2547
Mm-Aif1-C4	biotechne	cat# 319141-C4
Mm-Fosl1	biotechne	cat# 421981
Mm-HGF-C3	biotechne	cat# 315631-C3
Mm-Met-C2	biotechne	cat# 405301-C2

REAGENT or RESOURCE	SOURCE	IDENTIFIER
**Biological samples**		
Primary mouse glioma tissue	this paper	N/A

REAGENT or RESOURCE	SOURCE	IDENTIFIER
**Cell lines**		
DF-1	ATCC	cat# CRL-12203
GL261	Szatmari et al., 2006	RRID: CVCL_Y003
Human umbilical vein endothelial cells	ThermoFisher Scientific	cat# C0035C
Mouse bone marrow derived macrophages	primary culture	N/A
Mouse brain vascular pericytes	iXCells Biotechnologies	cat# 10MU-014
p53$^{-/-}$, *H-Ras* over-expressing	Hertwig et al., 2012	N/A

REAGENT or RESOURCE	SOURCE	IDENTIFIER
**Chemicals, peptides and recombinant proteins**		
Accumax	Sigma-Aldrich	cat# A7089
B27	ThermoFisher Scientific	cat# 17504044
Bioanalyzer High Sensitivity Chip	Agilent Technologies	cat# 5067-4626
Cell strainer	VWR	cat# 734-0002
DMEM high glucose	Corning	cat# 10-013-CMR
DMEM/F12	Corning	cat# 10-014-CV
DMEM/F12 with Glutamax	Gibco	cat# 10565018
Endothelial Cell Medium MV	PromoCell	cat# C-22020
Fetal Bovine Serum (FBS)	Corning	cat# 35-015-CV
Glutamine	Corning	cat# 25-005-CI
Green (Oregon) Dextran, 70kDa	ThermoFisher Scientific	cat# D7173
hEGF	R&D	cat# 236-EG-200
Human Pericyte Medium	3H Biomedical	cat# SC1201
Hypoxyprobe™ Kit	hpi	cat# HP1-XXX
IVISbrite D-Luciferin Bioluminescent Substrate in RediJect Solution (XenoLight)	Revvity	cat# 770504
KAPA SYBR FAST qPCR master mix	Sigma Aldrich	cat# KK4618
Mouse M-CSF	Miltenyi	cat# 130-101-704
Mouse rHGF	R&D	2207-HG-025
Mouse rGAS6	R&D	986-GS-025
Murine Recombinant IFNγ	PeproTech	cat# 315-05
Murine Recombinant IL-4	PeproTech	cat# 214-14
OCT Cryomount	Histolab	cat# 45830
Opal 480 reagent pack	Akoya Biosciences	cat# FP1500001KT
Opal 520 reagent pack	Akoya Biosciences	cat# FP1487001KT
Opal 570 reagent pack	Akoya Biosciences	cat# SKU FP1488001K

Opal 620 reagent pack	Akoya Biosciences	cat# SKU FP1495001K
Opal 690 reagent pack	Akoya Biosciences	cat# SKU FP1497001K
Opal 780 reagent pack	Akoya Biosciences	cat# FP1501001KT
Penicillin/Streptomycin	Corning	cat# 30-001-CI
rhFGF (10 ng/µl)	R&D	cat# 233-FB-025
RPMI 1640	Corning	cat# 10-043-CVR
Serum-free blocking reagent	DAKO	cat# X090930-2

REAGENT or RESOURCE	SOURCE	IDENTIFIER
**Critical commercial assays**		
Adult Brain Dissociation Kit, Mouse and Rat	Miltenyi	cat# 130-107-677
MycoAlert™ Mycoplasma Detection Kit	Lonza	cat# LT07-318
NovaSeq 6000 S1 Reagent Kit	Illumina	cat# 20028319
RNA-Protein Co-Detection Ancillary Kit	biotechne	cat# 323180
RNAscope 4-plex Ancillary Kit for Multiplex Fluorescent Reagent Kit v2	biotechne	cat# 323120
RNAscope Multiplex Fluorescent Reagent Kit v2	biotechne	cat# 323100
SPRIselect Reagent kit	Beckman Coulter	cat# B23318

REAGENT or RESOURCE	SOURCE	IDENTIFIER
**Plasmids**		
CMMP-luc	Laurent Roybon, Lund University	N/A

REAGENT or RESOURCE	SOURCE	IDENTIFIER
**Software and algorithms**		
Angiotool version 0.5	Zudaire et al., 2011	https://ccrod.cancer.gov/confluence/display/ROB2/Home RRID: SCR_016393
Celda (decontX) version 1.10.0	Campbell et al.	https://github.com/compbiomed/celda DOI:10.18129/B9.bioc.celda
Cell Ranger version 6.1.2	10x Genomics	https://www.10xgenomics.com/support/software/cell-ranger/latest RRID:SCR_017344
CellChat version 1.4.0	Jin et el., 2021	https://github.com/sqjin/CellChat RRID:SCR_021946
CellPhoneDB version 2.0.0	Efremova et al., 2020	https://www.cellphonedb.org/ RRID: SCR_017054
corrplot version 0.92	Wei and Simko, 2021	https://CRAN.R-project.org/package=corrplot RRID: SCR_024683
DoRothEA version 1.6.0	Garcia-Alonso et al., 2019 Badia-i-Mompel et al., 2022 Müller-Dott et al., 2023	https://bioconductor.org/packages/release/data/experiment/html/dorothea.html
DoubletFinder version 2.0.3	McGinnis et al. 2019	https://github.com/chris-mcginnis-ucsf/DoubletFinder https://www.perkinelmer.com/Content/LST_Software_Downloads/PhenochartWhatsNew_1_0_7_rev0.pdf RRID:SCR_018771

Ensembl release 111	EMBL-EBI	http://www.ensembl.org/<br>RRID:SCR_002344
Fiji version 2.9.0	Schindelin et al.,<br>2012	http://fiji.sc/<br>RRID: SCR_002285
FlowJo version 10.9.0	BD Biosciences	https://www.flowjo.com/solutions/flowjo<br>RRID: SCR_008520
ggpubr version 0.6.0	Alboukadel<br>Kassambara et al.,<br>2023	https://rpkgs.datanovia.com/ggpubr/<br>SCR_021139
GraphPadPrism<br>version 10.0.2	GraphPad	http://www.graphpad.com/<br>RRID: SCR_002798
Harmony version 1.0	Korsunsky et al., 2019	https://github.com/immunogenomics/harmon<br>y<br>RRID:SCR_022206
InForm version 2.4.10	Akoya Biosciences	https://www.perkinelmer.com/CMSResources/I<br>44-140143BRO_010576_01_PRD_inForm.pdf<br>RRID: SCR_019155
InterCellar version 2.0.0	Interlandi et al.,<br>2022	https://bioconductor.org/packages/release/bioc<br>nterCellar.html
Living Image Analysis<br>Software version 4.7.3	PerkinElmer	http://www.perkinelmer.com/catalog/category/i<br>%20image%20software<br>RRID: SCR_014247
Loupe Browser version 6.5.0.	10x Genomics	https://support.10xgenomics.com/single-cell-ge<br>expression/software/visualization/latest/what-is<br>cell-browser<br>RRID: SCR_018555
Metascape<br>version 3.5.20240101	Zhou et al., 2019	http://metascape.org/gp/index.html - /main/ste<br>RRID: SCR_016620
MiloR version 1.10.0	Dann et al., 2022	https://github.com/MarioniLab/miloR
Phenochart version 1.0.12	Akoya Biosciences	https://www.perkinelmer.com/Content/LST_Sof<br>Downloads/PhenochartWhatsNew_1_0_7_rev0.<br>RRID: SCR_019156
Qupath version 0.4.3	Bankhead et al.,<br>2017	http://qupath.github.io/<br>RRID: SCR_018257
R version 4.3.3	R project for Statistical<br>Computing	http://www.r-project.org/<br>RRID:SCR_001905
ScCustomize version 2.1.2	Marsh et al., 2021	https://doi.org/10.5281/zenodo.5706430<br>SCR_024675
SCpubr version 2.0.2	Blanco-Carmona<br>et al., 2022	https://www.biorxiv.org/content/10.1101/2022.0<br>82303v1
sctransform version 0.4.1	Hafemeister et al.,<br>2019	https://cran.r-<br>project.org/web/packages/sctransform/readme/<br>ME.html<br>RRID: SCR_022146
Seurat version 4.0.3	Hao et al., 2021; Stuart et al., 2019; Butler et al., 2018; Satija et al., 2015	http://seurat.r-forge.r-project.org/<br>RRID:SCR_007322
Space Ranger version 2.0.1	10x Genomics	https://www.10xgenomics.com/support/softwa<br>e-ranger/latest<br>RRID: SCR_017344
Spacexr<br>version 2.2.1 (RCTD)	Cable, et al., 2021	https://github.com/dmcable/spacexr
SPOTlight version 1.6.3	Elosua-Bayes et al., 2021	https://github.com/MarcElosua/SPOTlight
survival version 3.5-5	Thernau et al.,<br>2024	https://github.com/therneau/survival<br>SCR_021137
survminer version 0.4.9	Kassambra et al., 2021	https://rpkgs.datanovia.com/survminer/index.ht<br>RRID: SCR_021094
Xenium Explorer 4.0.0	Manz et al., 2022	https://www.10xgenomics.com/support/software/<br>m-explorer/latest<br>RRID: SCR_025847
