# Supplementary material for: Pericytes orchestrate a tumor-restraining microenvironment in glioblastoma
Source: Nat Commun. 2025 Dec 4;16:10918. doi: 10.1038/s41467-025-66985-1 (PMC12680652; doi:10.1038/s41467-025-66985-1)
Supplement: Supplementary file 2 — Reporting Summary [file 41467_2025_66985_MOESM2_ESM.pdf]

nature portfolio

Corresponding author(s): Kristian Pietras

Last updated by author(s): Oct 11, 2025

Reporting Summary

Nature Portfolio wishes to improve the reproducibility of the work that we publish. This form provides structure for consistency and transparency in reporting. For further information on Nature Portfolio policies, see our Editorial Policies and the Editorial Policy Checklist.

Statistics

For all statistical analyses, confirm that the following items are present in the figure legend, table legend, main text, or Methods section.

n/a	Confirmed	
☐	☒	The exact sample size (*n*) for each experimental group/condition, given as a discrete number and unit of measurement
☐	☒	A statement on whether measurements were taken from distinct samples or whether the same sample was measured repeatedly
☐	☒	The statistical test(s) used AND whether they are one- or two-sided<br>*Only common tests should be described solely by name; describe more complex techniques in the Methods section.*
☒	☐	A description of all covariates tested
☐	☒	A description of any assumptions or corrections, such as tests of normality and adjustment for multiple comparisons
☐	☒	A full description of the statistical parameters including central tendency (e.g. means) or other basic estimates (e.g. regression coefficient) AND variation (e.g. standard deviation) or associated estimates of uncertainty (e.g. confidence intervals)
☐	☒	For null hypothesis testing, the test statistic (e.g. *F*, *t*, *r*) with confidence intervals, effect sizes, degrees of freedom and *P* value noted<br>*Give P values as exact values whenever suitable.*
☒	☐	For Bayesian analysis, information on the choice of priors and Markov chain Monte Carlo settings
☒	☐	For hierarchical and complex designs, identification of the appropriate level for tests and full reporting of outcomes
☐	☒	Estimates of effect sizes (e.g. Cohen's *d*, Pearson's *r*), indicating how they were calculated

Our web collection on statistics for biologists contains articles on many of the points above.

Software and code

Policy information about availability of computer code

Data collection	See "Experimental Procedures", section Key Resources Table/Software and algorithms.
Data analysis	All analyses were performed using open-source software and algorithms already described in the literature and they are listed in the supplementary information file. The pipeline for the analyses will be made available upon request. Custom code used in the manuscript is available at https://github.com/KPLab/Pericytes_GBM.

For manuscripts utilizing custom algorithms or software that are central to the research but not yet described in published literature, software must be made available to editors and reviewers. We strongly encourage code deposition in a community repository (e.g. GitHub). See the Nature Portfolio guidelines for submitting code & software for further information.

Data

Policy information about availability of data

All manuscripts must include a data availability statement. This statement should provide the following information, where applicable:

- Accession codes, unique identifiers, or web links for publicly available datasets
- A description of any restrictions on data availability
- For clinical datasets or third party data, please ensure that the statement adheres to our policy

The raw and processed (rds objects) scRNA-seq and Visium spatial transcriptomics data are available through the GEO repository with accession numbers GSE272236 and GSE272237 , respectively.

Research involving human participants, their data, or biological material

Policy information about studies with human participants or human data. See also policy information about sex, gender (identity/presentation), and sexual orientation and race, ethnicity and racism.

Reporting on sex and gender	*Use the terms sex (biological attribute) and gender (shaped by social and cultural circumstances) carefully in order to avoid confusing both terms. Indicate if findings apply to only one sex or gender; describe whether sex and gender were considered in study design; whether sex and/or gender was determined based on self-reporting or assigned and methods used. Provide in the source data disaggregated sex and gender data, where this information has been collected, and if consent has been obtained for sharing of individual-level data; provide overall numbers in this Reporting Summary. Please state if this information has not been collected. Report sex- and gender-based analyses where performed, justify reasons for lack of sex- and gender-based analysis.*
Reporting on race, ethnicity, or other socially relevant groupings	*Please specify the socially constructed or socially relevant categorization variable(s) used in your manuscript and explain why they were used. Please note that such variables should not be used as proxies for other socially constructed/relevant variables (for example, race or ethnicity should not be used as a proxy for socioeconomic status). Provide clear definitions of the relevant terms used, how they were provided (by the participants/respondents, the researchers, or third parties), and the method(s) used to classify people into the different categories (e.g. self-report, census or administrative data, social media data, etc.) Please provide details about how you controlled for confounding variables in your analyses.*
Population characteristics	*Describe the covariate-relevant population characteristics of the human research participants (e.g. age, genotypic information, past and current diagnosis and treatment categories). If you filled out the behavioural & social sciences study design questions and have nothing to add here, write "See above."*
Recruitment	*Describe how participants were recruited. Outline any potential self-selection bias or other biases that may be present and how these are likely to impact results.*
Ethics oversight	*Identify the organization(s) that approved the study protocol.*

Note that full information on the approval of the study protocol must also be provided in the manuscript.

Field-specific reporting

Please select the one below that is the best fit for your research. If you are not sure, read the appropriate sections before making your selection.

☒ Life sciences ☐ Behavioural & social sciences ☐ Ecological, evolutionary & environmental sciences

For a reference copy of the document with all sections, see nature.com/documents/nr-reporting-summary-flat.pdf

Life sciences study design

All studies must disclose on these points even when the disclosure is negative.

Sample size	Sample sizes were determined based on pilot experiments and standard practice in life sciences. No statistical power analysis was done.
Data exclusions	No data were excluded from the analyses.
Replication	To ensure reproducibility, experiments were done in triplicates and repeated with different cell lines, animal models and cohorts, respectively.
Randomization	Research animals were grouped based on genetic background, and sex-mixed.
Blinding	Experimental blinding was not considered relevant for this study as standardized test protocols, objective, quantifiable measures and statistical control methods were all applied. No performance observations or behavioral tests were done.

Reporting for specific materials, systems and methods

We require information from authors about some types of materials, experimental systems and methods used in many studies. Here, indicate whether each material, system or method listed is relevant to your study. If you are not sure if a list item applies to your research, read the appropriate section before selecting a response.

Materials & experimental systems

n/a	Involved in the study
☐	☒ Antibodies
☐	☒ Eukaryotic cell lines
☒	☐ Palaeontology and archaeology
☐	☒ Animals and other organisms
☒	☐ Clinical data
☒	☐ Dual use research of concern
☒	☐ Plants

Methods

n/a	Involved in the study
☒	☐ ChIP-seq
☐	☒ Flow cytometry
☒	☐ MRI-based neuroimaging

Antibodies

Antibodies used	See "Supplementary information file", section Key Resources Table/Antibodies and probes.
Validation	Each antibody applied in this study was chosen based on the availability and quality of data on the usage of the respective antibody in similar experimental settings. Manufacturers list in detail references for each used antibody (see manufacturers websites). Details about suppliers can be found in the "Supplementary information file", section Key Resources Table/Antibodies and probes.

Eukaryotic cell lines

Policy information about cell lines and Sex and Gender in Research

Cell line source(s)	See "Supplementary information file", section section Key Resources Table/Cell lines.
Authentication	DF1 cells used in this study are virus generating cells and were not functionally scrutinized. Mouse bone marrow derived macrophages, mouse brain vascular pericytes (iXCells, Lot#200639) and p53-/-, H-Ras overexpressing mouse glioma cells are primary cells. HUVEC cells are are certified by Thermo Fisher Scientific and Gl261 cells are certified by IDEXX BioResearch. Authentication is provided. Established cell lines were validated by genetic profiling. A panel of microsatellite markers was used for PCR based genotyping.
Mycoplasma contamination	All cell lines used in this study tested negative for mycoplasma contamination in routinely done screenings.
Commonly misidentified lines (See ICLAC register)	No cell lines registered with ICLAC were used in conducting this study.

Animals and other research organisms

Policy information about studies involving animals; ARRIVE guidelines recommended for reporting animal research, and Sex and Gender in Research

Laboratory animals	Mus musculus; mixed background including C57BL/6 and FVB; 3-9 months old. Mice were kept under controlled temperature and humidity with a consistent light-dark cycle (12:12 hours), minimal noise and vibration exposure, standard caging, suitable bedding and free access to sterilized water and diet. Pdgfbret/ret mice (Lindblom et al., 2003) are bred on a C57BL/6 back-ground and were kindly provided by Christer Betsholtz (Ka-rolinska Institutet, Stockholm). Ntv-a mice (Squatrito et al., 2010) are bred on a mixed background including C57BL/6 and FVB and were kindly provided by Massimo Squatrito (Spanish National Cancer Research Center, Madrid). Mice used for experiments were 3-9 months old.
Wild animals	The study did not involve wild animals.
Reporting on sex	All cohorts were balanced with regard to sex.
Field-collected samples	The study did not involve samples collected from the field.
Ethics oversight	Malmö/Lund Ethics Committee on Animal Testing at the Lund District Court (permit numbers M167/15, 14122/2020); veterinaries at Lund University Centre for Comparative Medicine (CCM)

Note that full information on the approval of the study protocol must also be provided in the manuscript.

Plants

Seed stocks

Report on the source of all seed stocks or other plant material used. If applicable, state the seed stock centre and catalogue number. If plant specimens were collected from the field, describe the collection location, date and sampling procedures.

Novel plant genotypes

Describe the methods by which all novel plant genotypes were produced. This includes those generated by transgenic approaches, gene editing, chemical/radiation-based mutagenesis and hybridization. For transgenic lines, describe the transformation method, the number of independent lines analyzed and the generation upon which experiments were performed. For gene-edited lines, describe the editor used, the endogenous sequence targeted for editing, the targeting guide RNA sequence (if applicable) and how the editor was applied.

Authentication

Describe any authentication procedures for each seed stock used or novel genotype generated. Describe any experiments used to assess the effect of a mutation and, where applicable, how potential secondary effects (e.g. second site T-DNA insertions, mosiacism, off-target gene editing) were examined.

Flow Cytometry

Plots

Confirm that:

☒ The axis labels state the marker and fluorochrome used (e.g. CD4-FITC).

☒ The axis scales are clearly visible. Include numbers along axes only for bottom left plot of group (a 'group' is an analysis of identical markers).

☒ All plots are contour plots with outliers or pseudocolor plots.

☒ A numerical value for number of cells or percentage (with statistics) is provided.

Methodology

Sample preparation

See "Experimental Procedures", section FACS.

Instrument

BD FACS Melody Cell Sorter Ref. #661938

Software

FACSChorus Version 3.0, Becton Dickinson 2016 (analysis); FlowJo 10.9.0 Becton Dickinson (post-analysis)

Cell population abundance

The sort purity was validated by re-anlysis and determined to >80% target population enrichment.

Gating strategy

Gating for M2-like Mɸ: main population->singlet cells->living cells->F4/80+ cells->CD11B+,CD206+ cells.
Gating for MDSC: main population->singlet cells->living cells->LY6G- cells->CD11B+, LY6C+ cells.
Gating for CD45+: main population->singlet cells->living cells->CD45+
Gating for CD45+,CD3+,CD4+,CD8-: main population->singlet cells->living cells->CD45+,CD3+->CD4+,CD8-
Gating for CD45+,CD3+,CD4-,CD8+: main population->singlet cells->living cells->CD45+,CD3+->CD4-,CD8-
Gating for CD45+,CD3+,CD4-,CD8-: main population->singlet cells->living cells->CD45+,CD3+->CD4-,CD8-
Gating for CD45+,CD19+: main population->singlet cells->living cells->CD45+->CD19+
Gating for CD45+,NKP46+: main population->singlet cells->living cells->CD45+->NKP46+
Gating for CD45+,CD11b+: main population->singlet cells->living cells->CD45+,CD11b+
Gating for CD45+,CD11b+,Ly6C+,CD64+: main population->singlet cells->living cells->CD45+,CD11b+->Ly6C+,CD64+
Gating for CD45+,CD11b+,Ly6C+,CD64-: main population->singlet cells->living cells->CD45+,CD11b+->Ly6C+,CD64-
Gating for CD45+,CD11b+,Ly6C-,CD64+: main population->singlet cells->living cells->CD45+,CD11b+->Ly6C-,CD64+
Gating for CD45+,CD11b+,Ly6C-,CD64-,CD11c+,MHCII+: main population->singlet cells->living cells->CD45+,CD11b+->Ly6C-,CD64-->CD11c+,MHCII+

☒ Tick this box to confirm that a figure exemplifying the gating strategy is provided in the Supplementary Information.
